# Antibacterial Activity of *Zanthoxylum chalybeum*, *Aloe deserti*, and *Zanthoxylum usambarense*

**DOI:** 10.1155/bmri/6610508

**Published:** 2026-07-29

**Authors:** Stephen Mugambi Maithulia, Careen Ihazano Chumba, John Muthini Maingi, David Ng’ang’a Mburu, Eliud Nyaga Mwaniki Njagi

**Affiliations:** ^1^ Department of Biochemistry, Microbiology and Biotechnology, Kenyatta University, Nairobi, Kenya, ku.ac.ke

## Abstract

Various Gram‐positive and Gram‐negative enteric pathogens are responsible for bacterial gastroenteritis, and this condition is commonly treated with antibiotics. But, the growing prevalence of antimicrobial resistance and drug‐associated toxicities has diminished the effectiveness of conventional drugs, prompting the need for alternative remedies, especially from medicinal plants. This study assessed the in vitro antibacterial potential of *Zanthoxylum chalybeum* and *Zanthoxylum usambarense* and *Aloe deserti*. Standard bacterial strains from the Kenya Medical Research Institute were employed for antibacterial assays. Antibacterial activities were determined by means of inhibition zone diameters, MICs, MBCs, and time‐kill kinetics. Methanol extracts from *Zanthoxylum chalybeum* and *Aloe deserti* had no antibacterial activity. In contrast, the methanolic extract of *Zanthxylum usambarense* exhibited antibacterial action in a concentration‐ and time‐dependent fashion, with very potent efficacy against *Bacillus cereus*, *Bacillus subtilis*, and *Staphylococcus aureus*, moderate against *Enterococcus faecalis*, and low against *Escherichia coli*. Time‐kill studies confirmed rapid bactericidal action against susceptible Gram‐positive bacteria. Phytochemicals detected include alkaloids, flavonoids, tannins, terpenoids, and glycosides, as well as trace and heavy metals. *Zanthoxylum usambarense* exhibited remarkable antibacterial effect against Gram‐positive bacteria, but limited activity was exhibited against Gram‐negative strains. However, the present study was limited to in vitro testing, and post‐extraction quality control testing was not included. Thus, clinical application of the results cannot be made. Further studies are needed to identify the active constituents of the extract, toxicity, efficacy, and standardization of the extract.

## 1. Introduction

The most common etiologic agents responsible for bacterial gastroenteritis in developing countries include the enteric pathogens *Enterococcus faecalis*, *Bacillus subtilis*, *Staphylococcus aureus*, *Vibrio cholerae*, *Bacillus cereus*, *Escherichia coli*, *Salmonella typhi*, and *Shigella dysenteriae* [1]. Diarrhea is considered a very serious manifestation of this disease and mainly affects children [2] and elderly patients [3]. The antimicrobial resistance is a critical issue that is continuously growing as a global threat to human health. In 2023, one in every six laboratory‐confirmed cases of bacterial infections worldwide were resistant to antibiotics. Additionally, antimicrobial resistance increased in more than 40% of pathogen‐antibiotic pairs between 2018 and 2023. However, the distribution of antimicrobial resistance is not equal across the globe; higher antimicrobial resistance is seen in Africa and Asia than in Europe. Gram‐negative pathogens have the highest antimicrobial resistance to antibiotics such as third‐generation cephalosporins and carbapenems. In 2021, antimicrobial resistance caused 4.71 million deaths worldwide. Out of these deaths, 1.14 million were due to drug‐resistant infections. In 2050, antimicrobial resistance is predicted to result in increased deaths [3]. This growing resistance significantly undermines antimicrobial therapy, which remains the cornerstone of treatment, leading to prolonged illness, increased treatment costs, and a greater healthcare burden [4]. The problem is particularly severe in developing countries, where the misuse and overuse of antibiotics have accelerated the emergence of drug‐resistant bacteria, posing a serious public health challenge [5]. At the microbial level, antibiotic resistance arises through several adaptive mechanisms, including genetic mutations, reduced drug permeability, modification of target sites, and enzymatic inactivation of antimicrobial agents [6].

The main difference between Gram‐negative and Gram‐positive bacteria is the cell wall composition of these organisms [7]. Gram‐negative bacteria have a thin film of peptidoglycan and an outer membrane encapsulating lipopolysaccharides. Gram‐positive bacteria lack an outer membrane and are covered with a thick coating of peptidoglycan. Gram‐negative bacteria’s outer membrane operates as an antibiotic permeability barrier [8]. This is the rationale for Gram‐negative bacteria resistance to antibiotics. Gram‐negative bacteria are usually more difficult to treat, making them a priority in antimicrobial research.

Given the shortcomings of conventional antibiotics, especially against Gram‐negative bacteria, there has been a rising interest on plant‐derived chemicals as possible source of novel antibacterial medicines [9]. Herbal medicines have shown remarkable potential as alternative antibacterial agents against drug‐resistant bacterial pathogens. Several compounds from plants, such as benzoin, emetine, quinine, and berberine, have been used successfully in the treatment of infectious diseases, thereby justifying the place of phytomedicines in human health [10]. In developing countries, herbal medicines continue to be widely used because of their easy accessibility, affordability, and perceived lower toxicity than conventional antibiotics [11]. Increasing antibiotic resistance and toxicities associated with drugs have thus heightened interest in research on bioactive phytochemicals as alternative therapeutic agents. Various plant extracts have been previously reported to be effective against enteric pathogens, although most of them have shown stronger effect against Gram‐positive bacteria than Gram‐negative bacteria [12]. Such differences in susceptibility could be partly explained by the structural features of bacterial cell walls, where the outer membrane of Gram‐negative bacteria hinders the penetration of drugs and facilitates the enzymatic inactivation of drugs [13, 14]. Ethnobotanical knowledge has consistently contributed to the discovery of plant‐based antibacterial gents and has encouraged scientific verification of traditional medicinal treatments [15].

Local people in Meru County, Kenya utilize plants such as *Zanthoxylum chalybeum* and *Zanthoxylum usambarense* from *Rutaceae* family, and *Aloe deserti* from *Liliaceae* family for medical purposes. *Zanthoxylum chalybeum* is very common in the dry areas of Tigania West and is traditionally used for coughs, pneumonia, and gastrointestinal ailments [16]. *Aloe deserti*, a drought‐resistant perennial, is traditionally one of the most widely used plants in the management of malaria, wounds, and stomach disorders [17] while *Zanthoxylum usambarense* has gained widespread traditional uses for coughs, toothache, rheumatism, and gastrointestinal conditions [18]. This research was based on the hypothesis that the medicinal plant extracts chosen would possess antibacterial properties, with varying potency against Gram‐negative and Gram‐positive bacteria, and possibly enhanced potency against Gram‐negative bacteria.

## 2. Materials and Methods

### 2.1. Reagents and Chemicals

Mueller Hinton broth (Sigma‐Aldrich, Germany), chloroform, 95% methanol and DMSO (Schmidt Chemicals, Germany), Whatman no. 1 filter paper (Merck, Germany), anhydrous copper sulfate, nitric acid and hydrogen peroxide (Sigma‐Aldrich, Germany), distilled water, Bacl∙H_2_O (Merck, Germany), sulfuric acid, antibiotics ciproflaxin, and gentamycin (Glenmark Pharmaceuticals Ltd, India). The Organic Chemistry, Biochemistry, and Microbiology labs at Kenyatta University offered all the reagents and chemicals.

### 2.2. Plant Materials Collection

**Figure 1:**
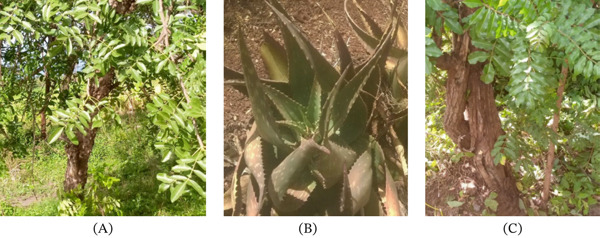
Representative images of the medicinal plants used in this study: (A) *Zanthoxylum chalybeum*, (B) *Aloe deserti*, and (C) *Zanthoxylum usambarense*. The images portray each species’ natural morphology and growth habits.

The plants collection was conducted under permit number NACOSTI/P/2323/5599 issued by the National Commission for Science, Technology and Innovation, in compliance with national regulations and ethical guidelines for plant research. Plant samples were gathered in Meru County’s Igembi North, Tigania West and Buuri subcounties. The locations used were *Zanthoxylum usambarense* (0.331067; 37.93679E; 1828.5 M), *Aloe deserti* (0.221158 N; 37.621957E; 1479.0 M), and *Zanthoxylum chalybeum* (0.021152 N; 37.636728; 1459.0 M). Plant samples were collected in January 2018, during the dry season, to maximize phytochemical yield. *Aloe deserti* is a perennial, acaulous, drought‐resistant plant that grows to about 100 cm on sandy soils. It spreads through offsets and produces terminal blooms that are white‐yellow in color. It contains mucilaginous jelly that serves as a water store during drought and has thick, clustered leaves with prickly borders. Only a few mature leaves (3–5) per plant were collected. *Zanthoxylum chalybeum* is a woody tree reaching about 12 m in height, with drooping branches spreading up to 1.3 m to form a canopy. Its dark stem and branches are covered with scattered thorns. The plant produces white terminal peduncle blooms and large, pointed bipinnate leaves with smooth margins. *Zanthoxylum usambarense* is a multi‐stemmed woody tree growing up to 14 m, with branches spreading up to 2 m to form a conical canopy. Its grayish‐brown stem is marked by straight, dark thorns, and it has broad, deep green bi‐pinnate leaves with smooth margins and creamy terminal flowers. The barks of both *Zanthoxylum chalybeum* and *Zanthoxylum usambarense* were collected from the outer layer using a sterilized knife, taking care not to damage the underlying wood. The sample from each plant was gathered at random and in triplicate. They were then completely combined and taken to the lab. The East African Herbarium in Kenya verified the plant barks and leaves. The vouchers SM01 (*Aloe deserti* A. Berger), SM02 (*Zanthoxylum chalybeum* var. *chalybeum*), and SM03 (*Zanthoxylum usambarense*) were placed in the Herbarium of the Department of Plant Sciences in Kenyatta. Photographs have been documented for verification (Figure 1).

### 2.3. Plant Materials Preparation

The fresh materials were cleaned to remove any unnecessary substances, then dried at room temperature for 2 weeks. The chopped materials were processed separately in Kenyatta University’s Animal Breeding Laboratory, using a Waring Blender for the leaves and a Wiley Mill, Model No. 2, United States, for the stem bark. Methanol was chosen as an extraction agent because it is capable of dissolving different water‐soluble and lipophilic compounds; it easily penetrates the plant cell walls to facilitate maximal release of compounds, resulting in high yields; and it rapidly evaporates after extraction, especially when using a rotary evaporator, thus facilitating concentration of the extract without exposing the extract to excessive heating that might compromise the integrity of heat‐sensitive compounds [19]. The fine powder from each plant (150 g) was transferred to a conical flask containing 1000 mL of methanol (1:6.7 w/v ratio) and shaken continuously for 24 h. The selected ratio was sufficient to ensure adequate solvent‐to‐solid interaction for efficient recovery of phytochemicals by preventing saturation of methanol and aiding in adequate immersion. The supernatants were then filtered through Whatman No. 1 filter paper and kept in a clean conical flask. A rotary evaporator (Bibby RE 100) operating at a reduced pressure of 150 mbar and a speed of 80 rpm was used to evaporate methanol to obtain crude extracts at 40°C. The pastes were transferred to vials and placed in an evacuated desiccator with anhydrous copper sulfate to dehydrate the extracts. The percentage yield of each plant extract was calculated based on the dry weight of the crude extract relative to the initial plant material. Each yield is reported as a single value (%). The extracts were weighed, yielding 16% (w/w) for *Zanthoxylum usambarense*, 12% (w/w) for *Aloe deserti*, and 15% (w/w) of *Zanthoxylum chalybeum* and then stored in the refrigerator at −20°C.

### 2.4. Qualitative Phytochemical Analysis

Secondary metabolites, including flavonoids, alkaloids, saponins, reducing sugars, terpenoids, anthraquinones, tannins, cardiac glycosides and in the methanol extracts were determined using the following standard qualitative methods with slight modifications. [20–25]. The presence of each metabolite was recorded using the following scoring system: − (absent), + (low), ++ (moderate), and +++ (high) based on intensity of color change or precipitation. All tests were conducted in triplicate to ensure reproducibility. Reagent blanks were used as negative controls to validate observed color changes. All analyses were conducted using standard laboratory glassware and a benchtop centrifuge. Given that this work was intended as an initial screening of antibacterial activity, qualitative phytochemical approaches were deemed adequate for identifying main compound groups. However, advanced analytical methods like and mass spectroscopy and high performance liquid chromatography need to be employed for precise identification purposes and should be considered in future studies.

#### 2.4.1. Alkaloid Test

1One percent hydrochloric acid (1.5 mL) was mixed with the plant extract (0.5 g) and then filtered. After adding Dragendorff’s reagent (potassium bismuth iodide solution), alkaloids were detected by the production of a reddish‐brown precipitate.

#### 2.4.2. Anthraquinones Test

Three milliliters of benzene was combined with 0.5 g of the plant extract and filtered. Five milliliters of a 10% ammonia solution was added to the filtrate and agitated. A pink, crimson, or violet tint in the ammonia (lower) phase showed the presence of anthraquinones.

#### 2.4.3. Saponins Test

Five milliliters of distilled water was mixed with 0.5 g of the methanolic plant extract and shaken vigorously. The mixture was mixed with three drops of olive oil. The creation of a stable emulsion was an indicator of saponins.

#### 2.4.4. Terpenoids Test

About 0.5 g of the plant extract was dissolved in 2 mL of chloroform. Then, 3 mL of sulfuric acid was gently dripped along the side of a test tube, which created a distinct layer from the solution. The appearance of a reddish‐brown coating suggested the presence of terpenoids.

#### 2.4.5. Tannin Test

In a test tube, 0.5 g of the plant extract was combined with 5 mL of water and filtered. The filtrate was then treated with three drops of 1% ferric chloride. Tannins were detected by the development of a brownish‐green color.

#### 2.4.6. Reducing Sugar Test

About 0.1 gram of plant extract was mixed with 2 mL of water. After adding 2 mL of Felling’s reagent, the mixture was submerged in water for 3 min. The presence of reducing sugar was indicated by the orange or brown color.

#### 2.4.7. Flavonoid Test

About 1.5 mL of 2% sodium hydroxide solution was combined with 0.5 g of the plant extract. The addition of two drops of 1% hydrochloric acid resulted in a change from yellow to colorless, revealing the existence of flavonoids.

#### 2.4.8. Cardiac Glycosides Test

Two milliliters of glacial acetic acid and a few drops of a 5% ferric chloride solution were mixed with an aliquot of 5 mL from each plant extract solution. One milliliter of strong sulfuric acid was carefully added to the mixture. The presence of cardiac glycosides was shown by the formation of a brown ring at the intersection.

### 2.5. Estimation of the Levels of Metal in the Extracts

Metal analysis was performed using atomic absorption spectrophotometry (AAS) on a Model 210 VGP equipment in flame mode with air‐acetylene and nitrous oxide‐acetylene flames [26]. The instrumental conditions for each metal were as follows: Ca (*λ* = 422.7 nm, 3.5 mA, slit width 0.2 nm), Zn (213.9 nm, 5.0 mA, 0.2 nm), Cu (324.8 nm, 5.0 mA, 0.2 nm), Mg (285.2 nm, 5.0 mA, 0.5 nm), Cr (357.9 nm, 5.0 mA, 0.2 nm), and Pb (217 nm, 10 mA, 1.0 nm). The air and acetylene flow rates were maintained at 7.0 and 1.8, respectively. Herbal extract (0.5 g) was digested by adding 5 mL of HNO₃ and 1 mL H₂O₂ in a 100 mL Pyrex beaker heated to 95°C for 15 min, then cooled, filtered, and diluted to 50 mL with distilled water. Standard solutions (certified 1000 ppm standard solutions) were used for calibration of instruments. All glassware was treated with 20% HNO₃ followed by washing with distilled water. The digested herbal extract was tested for Ca, K, Zn, Fe, Cu, Cr, Mg, Mn, Sr, Hg, V, As, and Pb metals. Calibration was done using standard solutions and blanks to check the instrument performance. Replicate analysis was done four times and metal concentrations were determined as:

Amount of metal=C×D/W

where D = Dilution factor, C = Concentration, and W = Sample weight (*μ*g/g).

### 2.6. Bacteria for Bioassays

All experiments were done in vitro. This study included no human participants or animals.

The microorganisms utilized in the study (Table 1) were acquired from stock cultures at the Centre for Microbiology Research of the Kenya Medical Research Institute, Kenya, because of reduced sensitivity or resistance to antibiotics such as *β*‐lactams. Bacterial strains were streaked from stock onto Mueller–Hinton agar to ensure purity based on colony morphology and Gram staining. A single, isolated colony was then subcultured into nutrient broth and kept at 37°C for 24 h to establish an active culture prior to experimentation. Their resistance profiles are shown in Table 1.

**Table 1:** Standard microorganisms used in the study and their resistance profile.

Bacterial strains	Resistance profile
Gram‐positive	
*Enterococcus faecalis* ATCC 29912	Exhibits intrinsic resistance to low levels of aminoglycosides, clindamycin, cephalosporins, and lincosamides [27].
*Bacillus cereus* ATCC 10876	Generally resistant to penicillin G and ampicillin due to the generation of ‐lactamases [28].
*Staphylococcus aureus* ATCC 29213	Methicillin susceptible (MSSA); no known resistance to conventional antibiotics (susceptible to oxacillin and other drugs) [29].
*Bacillus subtilis* ATCC 6633	Low‐level intrinsic antibiotic resistance, like ampicillin and cefotaxime [30].
Gram‐negative	
*Escherichia coli ATCC 25922*	Standard susceptible reference strain; no significant acquired resistance [31].
*Salmonella typhi ATCC 19430*	Reference susceptible strain; no known acquired multidrug resistance [32].
*Shigella dysenteriae ATCC 13313*	Frequently associated with multidrug resistance (e.g., ampicillin, tetracycline) [33].

### 2.7. 0.5 McFarland Standard Preparation

The 0.5 McFarland standard was made under aseptic conditions by adding 0.5 mL of 1.175% barium chloride to 99.5 mL of 1% sulfuric acid while swirling continuously to ensure even mixing. All reagents were checked for expiry and prepared using sterile distilled water. The resultant suspension was poured into 7 mL screw‐cap tubes and vortexed vigorously. The pipettes were calibrated and the spectrophotometer was calibrated and verified according to the instructions of the manufacturer before use. The optical density was measured at 625 nm, and its value was recorded as 0.092. The meniscus level of all the tubes was noted, and the standards were stored in the dark. Before each use, the suspension was examined for clumping and mixed evenly. These yielded approximately 1.5 × 10^8^ CFU/mL cell density [34].

### 2.8. Paper Discs

Sterile paper discs were made from Whatman No. 1 filter paper using a typical hole punch with a 6 mm diameter. These were sterilized by autoclaving at 121°C for 15 min, followed by drying in aseptic conditions [34]. These were stored in sterile vials with screw caps, with approximately 20–30 paper discs in each vial, ready for use. All operations involving paper disc handling were performed in aseptic conditions. Flame‐sterile forceps were used to handle the paper discs, with all vials and forceps being sterilized before use to prevent any contamination. For the impregnation process, each disc was treated with a predetermined volume of the test extract (10 *μ*L), at a concentration of 1000 mg/mL. This concentration was employed to overcome diffusion limitations of crude extracts and ensure that active compounds reached detectable levels in the agar. This process was done at 37°C for 15 min under sterile conditions to dry the discs and remove the solvent before application. The created discs were then placed on the top of inoculated agar plates using sterile forceps. The discs were positioned horizontally, with a minimum spacing of 24 mm center‐to‐center, which is considered the minimum distance required to prevent zones of inhibition overlap. Each plate included an acceptable number of discs. Positive controls included commercially manufactured antibiotic discs containing 10 *μ*g gentamicin and 30 *μ*g ciprofloxacin. The setups were run three times to confirm that results are were consistent.

### 2.9. Bacterial Inoculation

The test strains were grown overnight at 37°C in nutrient broth. The bacterial solution was diluted to achieve the 0.5 McFarland standard (approximately 1.5 × 10^8^ CFU/mL). The cotton swab was dipped in the solution, extra fluid was removed from it, and the agar surface was swabbed uniformly in three directions to achieve even coverage, and utilized immediately to assure proper concentration [35].

### 2.10. Evaluation of Inhibition Activity

The diameter of inhibition zones obtained around the tested samples was used to determine their antibacterial properties. The following standards were applied: 6–11 mm = weak level of inhibition, 12–18 mm = moderate level of inhibition, and > 1 mm = strong level of inhibition. For precision, two independent investigators carried out the measurements blindly using digital calipers. Inhibition zones were measured along two perpendicular lines, and the results were averaged to give means of three independent determinations for each sample, together with the respective controls [35].

### 2.11. Assessment of Minimum Inhibitory Concentration (MIC) and Minimum Bactericidal Concentration (MBC) Values

MICs of the plant extract against the test microorganisms were determined by the broth microdilution technique, which follows the Clinical and Laboratory Standards Institute guidelines [36]. The extracts were serially diluted twice in 96 well u‐shaped microtiter plates at concentrations ranging from 6.25 to 100 mg/mL. Each plate well contained 100 *μ*L of diluted extract and 10 *μ*L of bacterial solution which had been standardized at 1.5 × 10^8^ CFU/mL. The plates were then incubated for 24 h at 37°C. The MIC was obtained as the lowest extract concentration able to inhibit all growth of bacteria completely. The MBC was determined as the lowest extract concentration, which could kill the bacteria. In order not to overestimate bactericidal action, 10 *μ*L of bacterial suspension was placed in new 96‐well plates and incubated at 37°C for 4 h [37]. These aliquots were streaked on nutrient agar using a sterilized wire loop and incubated at 37°C for 24 h. The lack of bacteria on agar showed bactericidal activity whereas growth showed bacteriostatic activity. In order to achieve consistency, all experiments were performed thrice.

### 2.12. Assay for Time‐Kill

Time‐kill studies were done in order to assess the kinetics of antibacterial action of the plant extracts on selected bacterial strains. Standardized bacterial suspensions (~1.5 × 10^6^ CFU/mL, adjusted to McFarland standard) of *Bacillus cereus* ATCC 10876, *Bacillus subtilis* ATCC 6633, *Staphylococcus aureus* ATCC 29213, *Enterococcus faecalis* ATCC 29912, and *Escherichia coli* ATCC 35218 were used. Plant extracts were prepared in Mueller–Hinton broth in concentrations of 0.5× MIC, 1× MIC, and 2× MIC. One milliliter of standardized bacteria were added to 9 mL of plant extract solution and allowed to incubate in 37°C at 100 rpm. Samples were aseptically taken at different intervals (0, 1, 2, 3, 4, 5, 6, 12, and 24 h) and diluted 10 times using sterile normal saline. One hundred microliters of diluted sample was inoculated onto nutrient agar plates in triplicate and incubated in 37°C for 24 h. A number of viable organisms was calculated as cfu/plates. Bacterial density was expressed as colony‐forming units per milliliter (CFU/mL) using the standard formula:

CFUmL=Number of colonies×dilution factorVolume plated mL

All experiments were carried out in triplicate and results presented as mean ± SD. The data were then converted into log₁₀ CFU/mL in the determination of the kill kinetics. A bactericidal effect was considered if there was a ≥ 3 log₁₀ reduction in CFU/mL compared with the initial inoculum while bacteriostatic effect was determined if there was no ≥ 3 log₁₀ reduction in CFU/mL [38]. The regrowth after the first decrease in CFU/mL was considered as bacterial persistence or adaptation to the extract. Bacterial suspension that was not treated was used as the positive control while the negative control was the extract broth. The absence of any colony was taken as 0 CFU/mL and plotted on a logarithmic scale for graphical representation.

The change was presented in this way:

Percentage reduction=N0−NtN0

*N*_0_ represents the initial CFU/ML at Time 0.*N*_*t*_ represents CFU/mL at time *t* for every treatment.

While, the Log reduction was computed as:

Log reduction=Log1010N0−LogNt

### 2.13. Statistical Analysis

The experiment was conducted in triplicate, and results have been expressed as means ± standard deviation (SD). The number of samples was calculated according to the standard guidelines that must be followed during antibacterial susceptibility testing experiments. Normality and homogeneity of variance were determined using the Shapiro–Wilk and Levene’s tests, respectively. The analysis of statistics was carried out using [SPSS version 29/GraphPad Prism version 8]. One‐way ANOVA and Tukey’s HSD were used for group comparison tests and determining the significance of differences between treatment groups, respectively. A *p* value < 0.05 indicated a statistically significant difference. Outliers were assessed using Dixon Q method and no significant outliers were excluded unless justified by experimental error [39]. Although a formal a priori power calculation was not performed, the use of triplicate independent experiments provided sufficient statistical power to detect meaningful differences between groups, consistent with similar antibacterial studies [40].

## 3. Results

### 3.1. Percentage Yield and Phytochemical Profile

The methanol extract of *Zanthoxylum usambarense* produced 24 g (16%), *Aloe deserti* yielded 18 g (12%) and Z*anthoxylum. chalybeum* produced 15 g (10%). The highest amount of bioactive compounds found in *Zanthoxylum usambarense* plant extract included alkaloids, flavonoids, glycosides, tannins, and terpenoids. There were moderate quantities of reducing sugars and anthraquinones, but no saponins were detected. *Zanthoxylum chalybeum* contained significant tannins as well as moderate levels of flavonoids, alkaloids, glycosides, and anthraquinones. The analysis revealed low quantities of reducing sugars and terpenoids, but no saponins. The methanol extract of the *Aloe deserti* plant lacked flavonoids and saponins but did contain moderate tannins and low quantities of terpenoids, alkaloids, reducing sugars, glycosides, and anthraquinones (Table 2).

**Table 2:** Qualitative phytochemical screening of the extracts.

Phytochemicals	*Zanthoxylum chalybeum*	*Zanthoxylum usambarense*	*Aloe deserti*
Reducing sugars	+	++	+
Terpenoids	+	+++	+
Alkaloids	++	+++	+
Flavonoids	++	+++	—
Saponins	—	—	—
Cardiac glycosides	++	+++	+
Anthraquinones	++	++	+
Tannins	+++	+++	++

*Note:* +++ (high), ++ (moderate), + (low), — (Absent).

#### 3.1.1. Analysis of Trace Metals

Heavy metals and traces were measured through AAS. These findings (Table 3) depict that there are various macro‐ and micro‐nutrients, as well as heavy toxic metals in *Zanthoxylum usamberense*, *Zanthoxylum chalybeum*, and *Aloe deserti*, which are usually used for gastroenteritis treatment.

**Table 3:** Trace metals identified in the plant extracts.

Elements (*μ*g/g)	*Zanthoxylum chalybeum*	*Zanthoxylum usambarense*	*Aloe deserti*
Magnesium	144.00 ± 0.20	123.00 ± 0.05	111.91 ± 0.23
Manganese	4.13 ± 0.04	2.34 ± 0.05	3.25 ± 0.05
Calcium	37.71 ± 0.43	28.78 ± 0.24	22.02 ± 9.34
Potassium	45.00 ± 0.62	33.00 ± 2.55	43.00 ± 3.00
Zinc	2.44 ± 0.37	0.94 ± 0.34	3.90 ± 0.42
Iron	2.02 ± 0.08	1.45 ± 0.08	1.32 ± 0.54
Chromium	0.94 ± 0.16	0.03 ± 0.06	0.10 ± 0.15
Lead	0.06 ± 0.03	0.02 ± 0.03	BDL
Cupper	0.27 ± 0.07	BDL	0.42 ± 1.41
Strontium	0.11 ± 0.83	0.24 ± 0.27	BDL
Arsenic	0.03 ± 0.17	0.05 ± 0.23	0.02 ± 0.05
Mercury	BDL	BDL	BDL
Vanadium	BDL	BDL	BDL

*Note:* Results are shown as mean ± standard deviation (*N* = 3).

Abbreviation: BDL: mineral levels below the limit of detection by AAS.

Macronutrients (potassium, magnesium, and calcium) and micronutrients (manganese, zinc, copper, and iron) existed in all three plants. Trace heavy toxic metals (lead, chromium, strontium, vanadium, mercury, and arsenic) were present, but their values were low. Vanadium and mercury were below the AAS detection limit (BLD) in all three plants; while lead was BLD in *Aloe deserti* and copper was BLD in *Zanthoxylum usamberense*.

### 3.2. In Vitro Antibacterial Assessment

**Figure 2:**
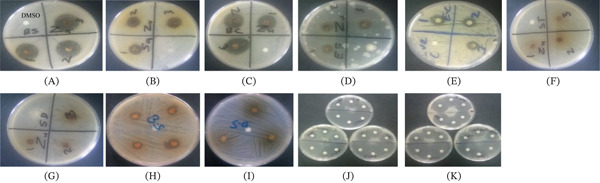
Growth inhibition of *Zanthoxylum usambarense* against (A) *Bacillus subtilis*, (B) *Staphylococcus aureus*, (C) *Bacillus cereus*, (D) *Enterococcus faecalis*, (E) *Escherichia coli*, (F) *Salmonella typhi*, and (G) *Shigella dysenteriae*. Growth inhibition of *Zanthoxylum chalybeum against* (H) B*acillus subtilis* and (I) *Staphylococcus aureus*. Growth inhibition of (J) Ciproflaxin and (K) Gentamycin.

**Figure 3:**
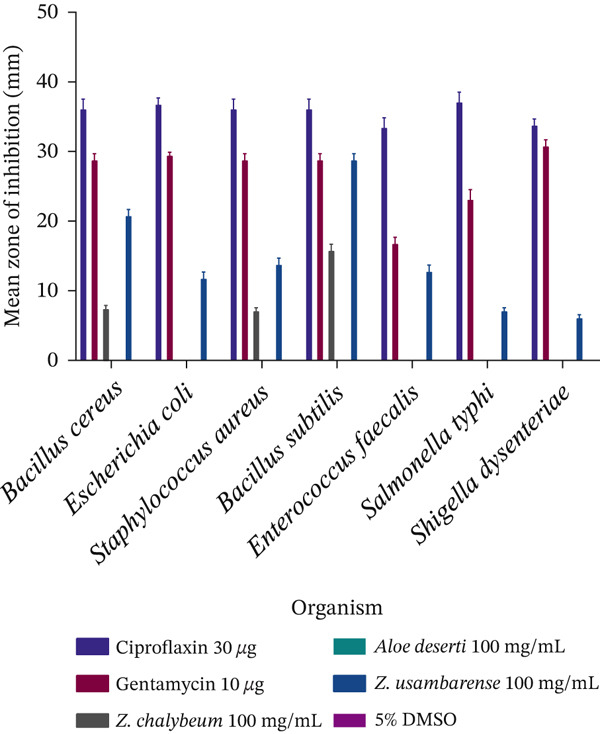
Antibacterial activity of plant extracts, showing the zone of inhibition against the tested organisms. Values are displayed as means ± standard deviation (*n* = 3).

An evaluation of antibacterial activity of these plant extracts was recorded in Table 4 and illustrated in Figures 2 and 3. Methanol extract of *Zanthoxylum usambarense* showed strong antibacterial activity against Gram‐positive bacteria, inhibiting *Bacillus cereus* and *Bacillus subtilis* strongly and *Staphylococcus aureus* moderately, while very weak inhibition was recorded against *Enterococcus faecalis*, *Salmonella typhi*, *Escherichia coli*, and *Shigella dysenteriae*. Methanol extract of *Zanthoxylum chalybeum* exhibited strong activity only against *Bacillus subtilis*, while the rest of the Gram‐positive bacteria exhibited a weak effect, and no Gram‐negative strains were active. No antibacterial activity was recorded for the *Aloe deserti* methanol extract. On the other hand, strong inhibitory activity against all tested bacteria was evident by ciprofloxacin and gentamicin; DMSO did not have any activity. Generally, Gram‐positive bacteria were more sensitive to plant extracts than Gram‐negative bacteria.

**Table 4:** Inhibition zones (mm) of plant extracts against test organisms.

Organisms	*Zanthoxylum chalybeum* (100 mg/mL)	*Aloe deserti* (100 mg/mL)	*Zanthoxylum usambarense* (100 mg/mL)	Ciprofloxacin (30 *μ*g)	Gentamycin (10 *μ*g)	5% DMSO
*Bacillus cereus* ATCC 10876	7.67 ± 1.25^∗^^,#^	NZ	22.67 ± 2.05^∗^^,#^	36.33 ± 1.25	29.00 ± 0.82	NZ
*Escherichia coli* ATCC 25922	NZ	NZ	12.00 ± 0.82^∗^^,#^	37.00 ± 0.82	29.67 ± 0.94	NZ
*Staphylococcus aureus* ATCC 29213	7.33 ± 0.47 ^∗^^,#^	NZ	14.00 ± 0.82^∗^^,#^	36.33 ± 1.25	29.00 ± 0.82	NZ
*Bacillus subtilis* ATCC 6633	16.00 ± 0.82^∗^^,#^	NZ	29.00 ± 0.82^∗^	36.33 ± 1.25	29.00 ± 0.82	NZ
*Enterococcus faecalis* ATCC 29912	NZ	NZ	13.00 ± 0.82^∗^^,#^	33.67 ± 1.25	17.00 ± 0.82	NZ
*Salmonella typhi* ATCC 19430	NZ	NZ	7.33 ± 0.47^∗^^,#^	37.33 ± 1.25	23.33 ± 1.25	NZ
*Shigella dysenteriae* ATCC 13313	NZ	NZ	6.33 ± 0.47^∗^^,#^	34.00 ± 0.82	31.00 ± 0.82	NZ

*Note:* Results are reported as means ± standard deviation (*n* = 3). Activity levels were categorized as follows: strong (> 18 mm), moderate (12–18 mm), and weak (6–11 mm).

NZ = no zone.

^∗^Indicates significant difference compared with negative control.

^#^Indicates significant difference compared with positive control (one‐way ANOVA, followed by Tukey’s post‐hoc test, *p* ≤ 0.05).

### 3.3. MIC, MBC Values, and the MBC and MIC Ratio for the Methanol Extract of *Zanthoxylum usambarense*

**Figure 4:**
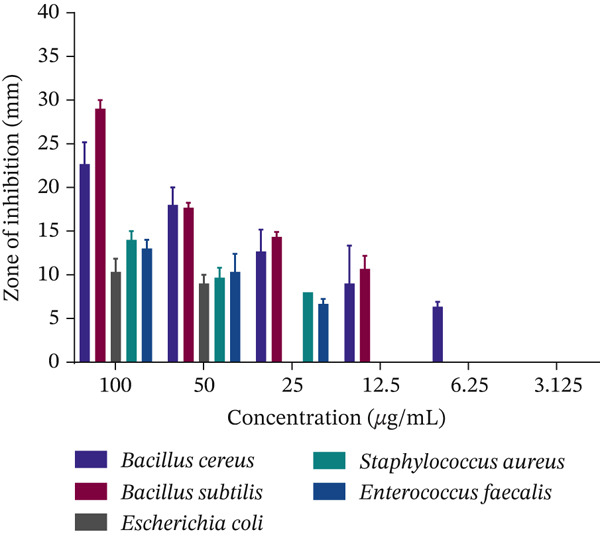
Inhibition zones (mm) of *Zanthoxylum usambarense* extract against selected bacterial strains at different concentrations (100–3.125 mg/mL). Values are displayed as mean ± standard deviation (*n* = 3).

Figure 4 shows inhibition zones obtained from the *Zanthoxylum usambarense* methanol extract against the tested microorganisms. The extract demonstrated good antibacterial activity against Gram‐positive bacteria with MICs of 6.5 mg/mL for *Bacillus cereus* and *Bacillus subtilis* and an MIC of 12.5 mg/mL for *Staphylococcus aureus*. MIC values were higher in *Escherichia coli* (50 mg/mL) and *Enterococcus faecalis* (25 mg/mL). Generally, *Bacillus cereus* and *Bacillus subtilis* showed significant sensitivity (*p* ≤ 0.05) to the extract at the lowest test concentration compared with other bacteria. The MBCs values were 12.5 mg/mL for *Bacillus subtilis*, 12.5 mg/mL for *Bacillus cereus*, 25 mg/mL for *Staphylococcus aureus*, 50 mg/mL for E*nterococcus faecalis*, and 100 mg/mL for *Escherichia coli*. The MBC/MIC ratio was 2.0 for *Escherichia coli*, *Bacillus cereus*, and *Enterococcus faecalis* and 1.0 for *Staphylococcus aureus* and *Bacillus subtilis* (Table 5).

**Table 5:** The MICs and MBC values of *Zanthoxylum usambarense* against test microorganisms.

Microorganism (ATCC)	Zone of inhibition at mg/ml (mm)							
	100	50	25	12.5	6.25	MIC (mg/mL)	MBC (mg/mL)	MBC/MIC
*Bacillus cereus* ATCC10876	22.67 ± 2.05^∗^	18.00 ± 1.63^∗^	12.67 ± 2.50^∗^	9.00 ± 1.25	6.33 ± 0.47	6.25	12.5	2.0
*Escherichia coli* ATCC25922	12.00 ± 0.82	9.00 ± 1.82	NZ	NZ	NZ	50	100	2.0
*Staphylococcus aureus* ATCC29213	14.00 ± 0.82^∗^	9.67 ± 0.94^∗^	8.00 ± 0.00	NZ	NZ	25	25	1.0
*Bacillus subtilis* ATCC 6633	29.00 ± 0.82^∗^	17.67 ± 047^∗^	14.33 ± 0.47^∗^	10.67 ± 1.45	NZ	12.5	12.5	1.0
*Enterococcus faecalis* 29912	13.00 ± 0.82^∗^	10.33 ± 0.17	6.67 ± 0.67	NZ	NZ	25.0	50	2.0

*Note*: Results are reported as means ± standard deviation (*n* = 3).

^∗^Indicates significant difference within the group (one‐way ANOVA, followed by Tukey’s post‐hoc test, *p* ≤ 0.05).

### 3.4. In Vitro Time‐Kill Kinetics of *Zanthoxylum usambarense* Extract

**Figure 5:**
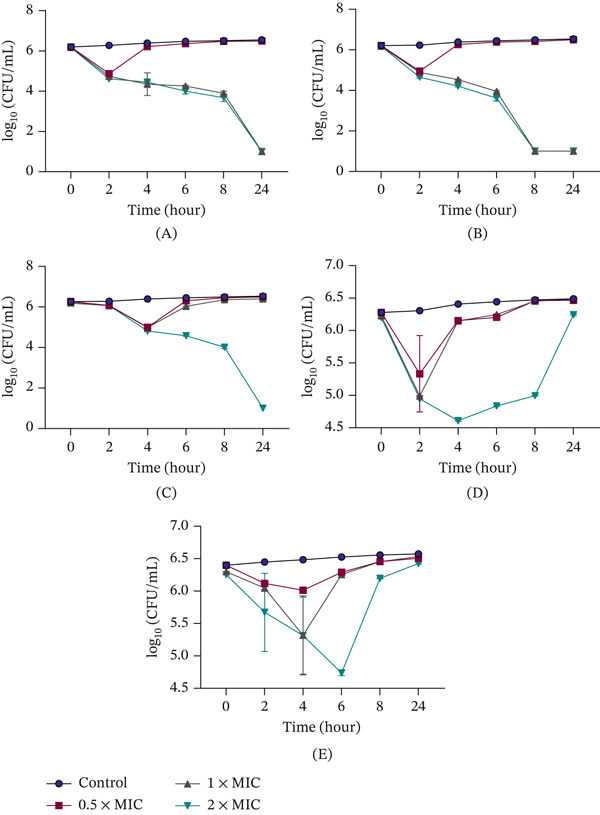
Time‐kill kinetics of various concentrations of *Zanthoxylum usambarense* against (a) *Bacillus cereus*, (b) *Bacillus subtilis*, (c) *Staphylococcus aureus*, (d) *Enterococcus faecalis*, and (e) *Escherichia coli*. Bacterial viability is expressed as log_10_ CFU/mL over time (0, 1, 2, 3, 4, 5, 6, 12, and 24 h). The *x*‐axis represents incubation time (hours), while the *y*‐axis represents log₁₀ CFU/mL. Different lines indicate treatment with plant extract at 0.5× MIC, 1× MIC, and 2× MIC, alongside an untreated control. Data represent mean ± standard deviation (*n* = 3). Values below the detection limit (10 CFU/mL) were plotted at 1 log_10_ CFU/mL.

Kinetics of *Zanthoxylum usambarense* extract against *Bacillus cereus* is provided in Table S1 and Figure 5. The initial population of bacteria was roughly equivalent between all groups (~10^6^ CFU/mL). Concentration‐dependent bacterial population reduction was recorded for the first 2 h, with percentage reductions between 53.0% and 71.2% (0.33–0.54 log_10_). The antibacterial property persisted up to 4 h for 1× MIC and 2× MIC concentrations, with percentage reductions of 68.2% and 80.5%, respectively, while there was regrowth in 0.5× MIC concentration. Strong antibacterial activity was observed after 6 h for 1× MIC and 2× MIC concentrations, with reductions more than 88%. Nearly complete cell elimination occurred by 24 h (> 99.9% reduction; 6.16 to 6.18 log_10_ reduction). However, regrowth occurred at 4 h for 0.5× MIC concentration with no subsequent bacterial inhibition. Control group continued to show an increase in the number of bacteria in all times tested *Zanthoxylum usambarense* extract was found to exhibit potent bactericidal effects against *Bacillus cereus* at 1× MIC and 2× MIC concentrations but not for 0.5× MIC concentration. The time‐kill kinetics of the effect of *Zanthoxylum usambarense* extract on *Bacillus subtilis* are illustrated in Table S2 and Figure 5. The initial bacterial inoculum level was similar for all test samples (~10^6^ CFU/mL). The first 2 h period shows a concentration‐related decrease in the number of bacteria with the percentage inhibition of bacterial growth of 45.2%–69.6% (0.26–0.51 log_10_). Inhibitory action became noticeable within 4 h with the percentage inhibition being 77.7% (at 1× MIC) and 89.0% (at 2× MIC) while regrowth was noted for 0.5× MIC. The antibacterial activity continued for 6 h and longer for both 1× MIC and 2× MIC with a decrease in bacterial numbers exceeding 94%. Almost complete killing of bacteria occurred during 8 h and was still observed at 24 h, with > 99.9% inhibition (reduction 6.17–6.18 log₁₀). Regrowth was observed at 4 h with no further inhibition noted for 0.5× MIC. The extract displayed potent bactericidal activity at 1× MIC and 2× MIC, but only transient effects at 0.5× MIC. Table S3 and Figure 5 show the time‐kill dynamics of *Zanthoxylum usambarense* extract on *Staphylococcus aureus*. Initial concentrations of bacteria for all treatments were comparable, at approximately 10^6^ CFU/mL. The percentage of reductions increased significantly for 4 h in all treatments, varying between 24.3% and 57.2% or log reductions of 0.12 and 0.37 log_10_. 2× MIC demonstrated the highest reduction rate during this time period. After the first 6 h, a clear concentration‐dependent effect started to emerge. While bacteria at 2× MIC concentration further decreased in concentration, with reductions reaching 93.2% at 8 h and > 99.9% at 24 h (concentrations <1.0 × 10^1^ CFU/mL), thus showing log reduction of 6.18; for lower concentrations, bacteria started to grow again after the first 6 h, with negative percentages of reductions shown for 8–24 h periods. Control group continued to show an increase in the number of bacteria in all times tested. Thus, the extract showed a strong bactericidal effect against *Staphylococcus aureus* at the 2× MIC concentration, while other concentrations displayed only short‐term inhibition. The time‐kill profile of *Zanthoxylum usambarense* extract on *Enterococcus faecalis* is summarized in Table S4. There was an initial decrease in bacterial population after 2–4 h of treatment in all concentrations, ranging from 41.7% to 74.3% (0.23–0.59 log_10_). The maximum effect was attained at 2× MIC after 4 h (74.3%; 0.59 log_10_), whereas there were minimal activities at other concentrations. As the experiment progressed (6–24 h), the antibacterial effect decreased, and regrowth was noted at 0.5× MIC and 1× MIC based on the percentage and log reductions that went below zero. Despite having an inhibitory effect until 8 h of treatment at 2× MIC, there was still regrowth by 24 h. Control group continued to show an increase in the number of bacteria in all times tested. The antibacterial effect of *Zanthoxylum usambarense* extract was time and concentration‐dependent without being bactericidal to *Enterococcus faecalis*. Time‐kill kinetics of *Zanthoxylum usambarense* against *Escherichia coli* is presented in Table S5 and Figure 5. Initial microbial population for all groups was almost identical (approximately 10^6^ CFU/mL). A decrease in microbial population was observed in all concentration treatments for the first 4 h, where percentage decrease ranged from 43.3% to 58.8% (0.25–0.39 log_10_). Percentage decrease was highest at 6 h at 2× MIC (69.4%; 0.52 log_10_) compared with other lower concentrations. However, a regrowth phenomenon was observed at both 8 and 24 h in all concentrations tested due to the negative percentage and log decreases. Control group continued to show an increase in the number of bacteria in all times tested. The extract exhibits a transient antibacterial activity with no bactericidal activity against *Escherichia coli*.

## 4. Discussion

The present study shows that methanolic extracts of Z*anthoxylum usambarense*, *Zanthoxylum chalybeum*, and *Aloe deserti* exhibit varying antibacterial activity that may be associated with differences in phytochemical composition. The percentage yields also varied, with *Zanthoxylum usambarense* recording the highest yield (16%), followed by *Aloe deserti* (12%) and *Zanthoxylum chalybeum* (10%). The yield of *Aloe deserti* is within the reported yields of other related species of *Aloe* genus, whereby methanol extract yields of *Aloe* species have been reported to range from approximately 12% [41] to over 20% [42], respectively. The difference in yield can be due to species variations, environmental factors, plant age, and method of extraction [43, 44].

In the case of *Zanthoxylum* species, the yields obtained have either matched or varied somewhat from those cited in earlier studies [16, 45], due to variations linked with parts of the plants, environmental factors, and extraction methods. [18]. Although stem bark is generally rich in secondary metabolites [46], factors such as geographical origin, seasonal variation, and post‐harvest handling can influence extraction efficiency [47]. Therefore, the yield differences observed in this study likely reflect methodological and biological variability rather than differences in phytochemical presence.

Phytochemical screening is often the first test performed on a material to determine the presence or absence of certain compound classes. This approach offers a qualitative answer, making it a useful initial evaluation tool [48]. The phytochemical analysis identified the presence of alkaloids, tannins, terpenoids, and glycosides in the plant extract, but the presence of flavonoids was exclusive to the *Zanthoxylum* genus. Similar results were discovered by a study by [16] who also did not find saponins in *Zanthoxylum Chalybeum* extract. In addition, Kaigongi et al. and Wekesa et al. [49, 50] reported alkaloids to be the major isolated compounds in the genus *Zanthoxylum*. On the contrary, some extracts of *Zanthoxylum chalybeum* [18] revealed the presence of saponins, which was not present in this study. The phytochemical profile of *Aloe deserti* observed in this study is largely consistent with reports from related *Aloe* species, particularly *Aloe vera*, which have been shown to contain anthraquinones, tannins, terpenoids, and glycosides [51]. However, the absence of certain phytochemicals such as saponins and flavonoids in this study contrasts with some previous reports [52]. These variations in phytochemical composition may be attributed to differences in the extraction solvent, the plant part used, the geographical origin, and the sensitivity of the qualitative screening methods [53].

Although both *Zanthoxylum usambarense* and *Zanthoxylum chalybeum* were extracted from stem bark, Z*anthoxylum. usambarense* had a higher phytochemical intensity (+++) in various categories such as alkaloids, terpenoids, flavonoids, and cardiac glycosides. This shows that changes in phytochemical strength are caused by differences between the two species (genus). Variations can be related to a variety of biological and ecological variables influencing secondary metabolite synthesis and concentration [54]. The solubility of methanol may also contribute to intensity changes [55]. The low phytochemical intensity and lack of flavonoids in *Aloe deserti* could be attributed to the inherent morphological and chemical features of *Aloe* leaf tissues. The parenchyma (leaf gel) is highly hydrated, being composed of about 98% water, and it is highly rich in polysaccharides; however, secondary compounds such as phenylpropanoids and flavonoids are common in the epidermis and latex regions of the leaf [56]. Recent metabolomic studies have revealed that flavonoids are unevenly distributed in *Aloe* leaves, with minimal or undetectable amounts in the gel fraction [57, 58]. As a result, extraction from gel‐dominated tissues might create minimal levels of phenolic compounds, resulting in low phytochemical intensity and the absence of flavonoids in qualitative assays.

All of the identified phytochemicals are known to have antibacterial effects. Tannin, alkaloid, terpenoids, and flavonoid‐containing plants have been shown to have antibacterial activity against Gram‐negative bacteria such as *Escherichia coli* and Gram‐positive bacteria such as *Bacillus cereus*, *Bacillus subtilis*, *Enterococcus faecalis*, and *Staphylococcus aureus* [59, 60], indicating that they function as a broad‐spectrum antibacterial agent. For example, benzophenanthridine alkaloids discovered in the *Zanthoxylum* genus can attach to bacterial DNA strands, blocking the enzyme against methicillin‐resistant *Staphylococcus aureus* [61]. The majority of the flavonoids identified in the *Zanthoxylum* genus are glycosides of flavonols, flavones, and flavanones such as quercetin, kaempferol, and isorhamnetin, which interfere with cellular membrane function and nucleic acid production [50, 62]. Tannins form irreversible complexes with bacteria’s proteins, interfering with metabolism [63]. Lupeol, a triterpenoid isolated from *Zanthoxylum chalybeum* [64] causes oxidative stress by generating nitric oxide, resulting in bacterial cell division arrest and apoptosis‐like death. It also has weak antibacterial activity against *Bacillus subtilis*, methicillin‐resistant *Staphylococcus aureus*, and *Escherichia coli* [65]. The antibacterial activities of anthraquinone mainly include inhibition of biofilm formation, cell wall destruction, and nucleic acid synthesis [66].

Elemental analysis showed that there were several macronutrients and micronutrients present in all plant extracts, but in different quantities. They include magnesium, calcium, potassium, iron, and zinc. The level of magnesium and calcium was higher in *Zanthoxylum chalybeum* than in the other plants, while the level of zinc was higher in *Aloe deserti* compared with others. Although certain minerals and heavy metals may possess poor antibacterial activities, secondary metabolites are often better for such purposes. Zinc and calcium, for example, have been linked to reduced microbial growth [67–69]. Notably, the plants with higher mineral concentrations, that is, *Zanthoxylum chalybeum* and *Aloe deserti*, had lower bacterial growth inhibition than *Zantoxylum usambarense*. As a result, the antibacterial characteristics in this case could be attributed to phytochemicals in the extracts, as mineral content is lower in the plant with significant bacterial growth inhibitory properties. Importantly, harmful heavy metals such as lead, arsenic, and mercury were found at extremely low quantities or below detectable levels, indicating that the plant materials fall within acceptable safety limits and may be useful for future pharmacological research.

*Zanthoxylum usambarense* exhibited inhibitory zones ranging from weak to strong activity (6.33–9.00 mm). The maximum activity was found against *Bacillus subtilis* (29.00 ± 0.82 mm), which falls within the strong inhibition group (> 18 mm), showing high susceptibility to the extract. Moderate to weak inhibition was found against *Bacillus cereus* (22.67 ± 2.05 mm), *Staphylococcus aureus* (14.00 ± 0.82 mm), *Enterococcus faecalis* (13.00 ± 0.82 mm), and *Escherichia coli* (12.00 ± 0.82 mm). The lowest inhibition zones were obtained against *Salmonella typhi* (7.33 ± 0.47 mm) and *Shigella dysenteriae* (6.33 ± 0.47 mm), demonstrating lower susceptibility of these enteric pathogens. The dissimilarity in resistance between Gram‐positive and Gram‐negative bacteria can be associated with structural differences between the two cell envelopes. Gram‐negative bacteria have an outer cell wall that is composed of lipopolysaccharide, thus preventing entry of phytochemicals into the cell while Gram‐positive bacteria lack this outer wall.

The antibacterial action of *Zanthoxylum chalybeum* was relatively poor since it only inhibited *Bacillus cereus* (7.67 ± 1.25 mm), *Staphylococcus aureus* (7.33 ± 0.47 mm), and *Bacillus subtilis* (16.00 ± 0.82 mm). Inhibitory effect on *Bacillus subtilis* was moderate while the other two bacteria were weakly inhibited. Inability to inhibit all Gram‐negative bacteria implies that the bioactive compounds in the plant extract may be insufficiently potent or present in insufficient quantities, or may inhibit structures specific to Gram‐positive bacteria. Earlier studies have demonstrated that the *Zanthoxylum chalybeum* extract exhibited effective antibacterial activity towards *Bacillus cereus* and *Staphylococcus aureus*, but mild antibacterial activity was observed against *Escherichia coli* [70] and poor activity against *Staphylococcus aureus* and *Escherichia coli* [71]. The reason for the difference in activity is because of the phytochemical variations and efficiency of extraction [72, 73].

The lack of any antibacterial zone in all strains of *Aloe deserti* is clearly distinctive from other species of *Aloes* that are often found to be antibacterial because of their presence of anthraquinones, flavonoids, and phenolics [74]. This may be attributed to differences in the phytochemical composition, environmental influence on metabolites production, or methanol solvent employed in the extraction process that cannot efficiently extract the active substances in this species [75]. The weak phytochemical activity of this plant can also justify the lack of observed biological activity. In addition, although *Aloe deserti* has been traditionally used for its therapeutic benefits, it showed no antibacterial effect. Such discrepancy may indicate that the therapeutic effects are not limited to antibacterial effects, but rather include anti‐inflammatory or immunomodulation effects, or that the bacteria tested were not the same targeted by traditional medicine. Moreover, the active compounds in *Aloe deserti* might need other extraction methods or might work better in vivo than in vitro.

Ciprofloxacin and gentamicin, the reference antibiotics, had significantly larger inhibition zones (29.00–37.33 mm), indicating that they were more potent than plant extracts. However, *Zanthoxylum usambarense* demonstrated activity comparable with gentamicin in some cases, particularly against *Bacillus subtilis* and *Bacillus cereus*. This demonstrates its potential not only as a traditional medicine, but also as a source of novel, potentially synergistic anti‐infective substances, which will be especially useful as the industry faces rising multidrug resistance.

According to the agar diffusion results, *Zanthoxylum usambarense* had the most consistent and broad‐spectrum antibacterial activity of the extracts. As a result, it was selected for additional research into its MIC, MBC, and time kill kinetics against five common bacterial strains in order to better describe its antibacterial activity.

*Zanthoxylum usambarense*’s antibacterial activity was clearly concentration‐dependent, with inhibition zones decreasing from 100 to 6.25 mg/mL across all sensitive species. The MIC values verified this pattern, with Gram‐positive bacteria having lower MICs (*Bacillus cereus*, 6.25 mg/mL; *Bacillus subtilis*, 12.5 mg/mL; *Staphylococcus aureus* and *Enterococcus faecalis*, 25 mg/mL) than *Escherichia coli* (50 mg/mL), demonstrating Gram‐positive species’ better susceptibility. MBC values were either equal to or twice as high as MICs, resulting in MBC/MIC ratios of 1.0 to 2.0. This reveals that the extract has bactericidal effect against *Staphylococcus aureus*, *Bacillus cereus*, *Bacillus subtilis*, *Escherichia coli*, and *Enterococcus faecalis* [42]. Previous study by Kaigongi et al. [76] discovered MIC values of 7.81 *μ*g/mL (*Staphylococcus aureus* and *Bacillus cereus*) and 15.63 *μ*g/mL (*Enterococcus coli*) for *Zanthoxylum usambarense*, 1.95 *μ*g/mL (*Staphylococcus aureus*), 3.91 *μ*g/mL (*Bacillus cereus*) and 7.81 *μ*g/mL (*Escherichia coli*) for *Zanthoxylum chalybeum*. From the above findings, it becomes evident that the tested extract exhibit limited antibacterial properties. The variation is mainly due to the type of extraction solvent used and the geographical source of the plant materials. These aspects determine the quality and quantity of phytochemical compounds.

The time‐kill kinetics of *Zanthoxylum usambarense* extract displayed a pronounced dose‐ and time‐dependent antibacterial activity in all tested strains, characterized by significant strain susceptibility variability. The rapid decrease of bacterial counts was observed in all strains within the first 2–4 h, thus indicating that the extract immediately inhibited bacterial growth. However, this effect was not consistently maintained. Certain organisms were able to regrow at further times, particularly at subinhibitory concentrations (0.5× MIC and, sometimes, 1× MIC). The bacteria that exhibited maximum sensitivity towards the test antibiotics were *Bacillus cereus* and *Bacillus subtilis*, with total destruction (decrease of > 99.9% at 1× MIC and 2× MIC in 8–24 h). Likewise, *Staphylococcus aureus* showed bactericidal activity, but at a higher concentration of 2× MIC. On the other hand, *Enterococcus faecalis* and *Escherichia coli* exhibited only transient inhibition of bacterial growth before regrowth was noted, suggesting that the phytochemicals acted as bacteriostatic. The regrowth noted in these cases may have been brought about by several factors, such as instability of the bioactive chemicals themselves, adaptation to the stress caused by the phytochemicals, or lack of sufficient concentrations to exert long‐term antibacterial activity. The presence of an outer membrane barrier, which prevents the diffusion of phytochemicals into the cell, could explain the relatively low susceptibility of *Escherichia coli* to the compounds tested.

Other than in vitro data, the antibacterial effect of *Zanthoxylum usambarense* extract holds promise for translational research purposes. The bactericidal effect against the species *Bacillus cereus*, *Staphylococcus aureus*, and *Bacillus subtilis* as well as the transient effect on *Escherichia coli* and *Enterococcus faecalis*, points to selective efficacy that can be enhanced using combined treatment. There have been studies that have shown how compounds derived from plants could help synergistically with conventional antibiotics in modifying resistance mechanisms such as efflux pumps or changes in membrane permeability thereby increasing antibacterial effects while reducing doses of antibiotics [77]. This proves that extract from *Zanthoxylum usambarense* could be used as an adjuvant for antibiotics. Further, whereas the present results are promising in terms of in vitro activity, it remains important to conduct in vivo studies to determine pharmacokinetics, toxicity, and efficacy in physiological conditions. There are multiple mechanisms by which plant extracts operate, including membrane disruption, enzyme inhibition, and antibiofilm properties, all of which may behave differently in complex biological systems [78]. With regard to public health issues, the emergence of antibiotic resistance has posed a serious global problem and calls for the development of new treatment approaches. Plants have been shown to contain compounds with multiple target effects as well as resistance modifying properties that can be used to combat resistance [79]. Therefore, further studies need to be conducted on the isolation and mode of action of the active components in *Zanthoxylum usambarense* as an antibiotic.

## 5. Conclusion

From this experiment, it can be concluded that *Zanthoxylum usambarense* extract has significant dose‐dependent and time‐dependent antibacterial activity, effectively achieving the study’s primary objective to screen and identify plants with antibacterial potential. The extract demonstrated strong bactericidal activity against Gram‐positive bacteria such as *Bacillus cereus*, *Bacillus subtilis*, and *Staphylococcus aureus* and moderate and temporary inhibition of growth of Gram‐negative bacteria like *Escherichia coli* and *Enterococcus faecalis*. Though the two plants used in the study did not have any antibacterial activity, the findings help limit further studies to the *Zanthoxylum* species alone. The unique aspect of the current study compared with routine disc diffusion experiments lies in its thorough examination of time‐kill dynamics against various bacteria that can be important for treatment. In describing the differences in sensitivity and characterizing the effects of extract on bacteria at specific periods of time, the current study gives a more comprehensive understanding of the antibacterial properties of the extract. These findings have significant public health implications; they suggest that *Zanthoxylum usambarense* contains bioactive compounds that could form a basis for natural alternatives against common human pathogens like *Staphylococcus aureus* and *Bacillus cereus*, which are major causes of foodborne illnesses and clinical infections, providing a critical weapon against the global threat of antibiotic resistance. Nonetheless, the experiment is limited to an in vitro experiment without any chemical isolation or toxicity studies. Future research needs to be done in the direction of in vivo testing, identification of bioactive compounds, and evaluation of their synergistic effects when combined with regular antibiotics.

## Author Contributions

Stephen Mugambi Maithulia contributed to the investigation, data analysis, and original draft writing. Careen Ihazano Chumba drafted the manuscript, edited, and revised the final draft. Eliud Nyaga Mwaniki Njagi, John Muthini Maingi, and David Ng’ang’a Mburu conceptualized, designed, supervised, and edited the original and final draft.

## Funding

The authors did not receive any funding for this research.

## Disclosure

All authors approved the final manuscript and agree to be accountable for the content and conclusions of the article.

## Ethics Statement

The authors have nothing to report.

## Conflicts of Interest

The authors declare no conflicts of interest.

## Supporting information


**Supporting Information** Additional supporting information can be found online in the Supporting Information section. Table S1: Time‐kill kinetics antimicrobial study of *Z. usambarense* extract against *Bacillus cereus*. Table S2: Time‐kill kinetics antimicrobial study of *Z. usambarense* extract against *Bacillus subtilis*. Table S3: Time‐kill kinetics antimicrobial study of *Z. usambarense* extract against *Staphylococcus aureus*. Table S4: Time‐kill kinetics antimicrobial study of Z. *usambarense* extract against *Enterococcus faecalis*. Table S5:Time‐kill kinetics antimicrobial study of Z. *usambarense* extract against *Escherichia coli.*

## Data Availability

The data that support the findings of this study are available on request from the corresponding author.
