# Supplementary material for: Antibacterial Activity of Zanthoxylum chalybeum, Aloe deserti, and Zanthoxylum usambarense
Source: Biomed Res Int. 2026 Jul 29;2026:6610508. doi: 10.1155/bmri/6610508 (PMC13420354; doi:10.1155/bmri/6610508)
Supplement: Supplementary file 1 — Supporting Information Additional supporting information can be found online in the Supporting Information section. Table S1: Time‐kill kinetics antimicrobial study of Z. usambarense extract against Bacillus cereus. Table S2: Time‐kill kinetics antimicrobial study of Z. usambarense extract against Bacillus subtilis. Table S3: Time‐kill kinetics antimicrobial study of Z. usambarense extract against Staphylococcus aureus. Table S4: Time‐kill kinetics antimicrobial study of Z. usambarense extract against Enterococcus faecalis. Table S5:Time‐kill kinetics antimicrobial study of Z. usambarense extract against Escherichia coli. [file BMRI-2026-6610508-s001.docx]

Supplementary material

Table S1. Time-kill kinetics antimicrobial study of Z. usambarense extract against Bacillus cereus

| Incubation time (Hour) | Mean bacterial population  (CFU/mL) | | | | Percentage reduction | | | Log reduction | | |
| --- | --- | --- | --- | --- | --- | --- | --- | --- | --- | --- |
|  | 0.5×MIC | 1×MIC | 2×MIC | Control | 0.5×MIC | 1×MIC | 2×MIC | 0.5×MIC | 1×MIC | 2×MIC |
| 0 | 1.58×10^6^±2.89^a^ | 1.51×10^6^±1.54^a^ | 1.45×10^6^±0.58^a^ | 1.58×10^6^±1.53^a^ | - | - | - |  |  |  |
| 2 | 7.43×10^5^±4.04^b^ | 5.50×10^5^±5.00^b^ | 4.17×10^5^±2.89^b^ | 1.89×10^6^±1.53^a*^ | 53.0 | 63.6 | 71.2 | 0.33 | 0.44 | 0.54 |
| 4 | 1.66×10^6^±5.29^*^ | 4.80×10^5^±2.00^b^ | 2.83×10^5^±3.21^c^ | 2.46×10^6^±1.00* | -5.1 | 68.2 | 80.5 | -0.02 | 0.50 | 0.71 |
| 6 | 2.30×10^6^±10.0^*^ | 1.80×10^5^±2.00^c^ | 1.03×10^5^±2.89^d^ | 30.0×10^6^±1.00^*^ | -45.6 | 88.1 | 92.9 | -0.16 | 0.92 | 1.15 |
| 8 | 3.00×10^6^±1.00^*^ | 8.00×10^4^±2.00^d^ | 5.00×10^4^±2.00^e^ | 3.21×10^6^±3.51^*^ | -89.9 | 94.7 | 96.6 | -0.28 | 1.28 | 1.46 |
| 24 | 3.11×10^6^±1.00^*^ | <1.0×10^1e^ | <1.0×10^1f^ | 3.55×10^6^±5.00^*^ | -96.8 | >99.9 | >99.9 | -0.29 | 6.18 | 6.16 |

Values are means ± standard deviation of three replicates. Values in each column followed by different superscript within each column are significantly different at p ≤ 0.05.Values followed by asterix indicate the regrowth of viable colony cells. < is below the detection limit.

Table S2. Time-kill kinetics antimicrobial study of Z. usambarense extract against Bacillus subtilis

| Incubation time (Hour) | Mean bacterial population  (CFU/mL) | | | | Percentage reduction | | | Log reduction | | |
| --- | --- | --- | --- | --- | --- | --- | --- | --- | --- | --- |
|  | 0.5×MIC | 1×MIC | 2×MIC | Control | 0.5×MIC | 1×MIC | 2×MIC | 0.5×MIC | 1×MIC | 2×MIC |
| 0 | 1.63×10^6^±1.00^a^ | 1.51×10^6^±2.00^a^ | 1.48×10^6^±2.00^a^ | 1.63×10^6^±2.00^a^ | - | - | - |  |  |  |
| 2 | 8.93×10^5^±9.09^b^ | 7.67×10^5^±7.64^b^ | 4.50×10^5^±5.00^b^ | 1.69×10^6^±2.00^a*^ | 45.2 | 49.2 | 69.6 | 0.26 | 0.29 | 0.51 |
| 4 | 1.82×10^6^±2.00^*^ | 3.37×10^5^±1.53^c^ | 1.63×10^5^±1.53^c^ | 2.42×10^6^±3.06* | -11.7 | 77.7 | 89.0 | -0.05 | 0.65 | 0.96 |
| 6 | 2.41×10^6^±3.06^*^ | 9.00×10^4^±1.00^d^ | 4.30×10^4^±1.53^d^ | 2.76×10^6^±1.53^*^ | -47.7 | 94.0 | 97.1 | -0.17 | 0.12 | 1.54 |
| 8 | 2.67×10^6^±3.06^*^ | <1.0×10^1e^ | <1.0×10^1e^ | 3.07×10^6^±2.52^*^ | -63.8 | >99.9 | >99.9 | -0.21 | 6.18 | 6.17 |
| 24 | 3.12×10^6^±3.06^*^ | <1.0×10^1e^ | <1.0×10^1e^ | 3.47×10^6^±4.16^*^ | -91.4 | >99.9 | >99.9 | -0.28 | 6.18 | 6.17 |

Values are means ± standard deviation of three replicates. Values in each column followed by different superscript within each column are significantly different at p ≤ 0.05.Values followed by asterix indicate the regrowth of viable colony cells. < is below the detection limit.

Table S3. Time-kill kinetics antimicrobial study of Z. usambarense extract against Staphylococcus aureus

| Incubation time (Hour) | Mean bacterial population  (CFU/mL) | | | | Percentage reduction | | | Log reduction | | |
| --- | --- | --- | --- | --- | --- | --- | --- | --- | --- | --- |
|  | 0.5×MIC | 1×MIC | 2×MIC | Control | 0.5×MIC | 1×MIC | 2×MIC | 0.5×MIC | 1×MIC | 2×MIC |
| 0 | 1.82×10^6^±2.00^a^ | 1.58×10^6^±2.00^a^ | 1.52×10^6^±1.73^a^ | 1.82×10^6^±1.00^a^ | - | - | - | - | - | - |
| 2 | 1.18×10^6^±2.52^a^ | 1.16×10^6^±2.31^a^ | 1.15×10^6^±5.00^a^ | 1.88×10^6^±2.65^a*^ | 35.3 | 26.4 | 24.3 | 0.19 | 0.13 | 0.12 |
| 4 | 1.00×10^6^±4.59^a^ | 9.67×10^5^±7.64^b^ | 6.50×10^5^±5.00^b^ | 2.44×10^6^±2.00* | 45.1 | 38.8 | 57.2 | 0.26 | 0.21 | 0.37 |
| 6 | 2.01×10^6^±2.31^*^ | 1.07×10^6^±1.53^*^ | 3.77×10^5^±2.52^c^ | 2.82×10^6^±5.86^*^ | -10.3 | 32.5 | 75.2 | -0.04 | 0.17 | 0.61 |
| 8 | 2.81×10^6^±3.06^*^ | 2.26×10^6^±4.04* | 1.03×10^5^±3.06^d^ | 3.10×10^6^±1.52^*^ | -54.2 | -42.8 | 93.2 | 0.19 | -0.16 | 1.17 |
| 24 | 3.08×10^6^±1.73^*^ | 2.50×10^6^±2.00* | <1.0×10^1e^ | 3.38×10^6^±4.58^*^ | -69.2 | -58.2 | >99.9 | -0.23 | -0.20 | 6.18 |

Values are means ± standard deviation of three replicates. Values in each column followed by different superscript within each column are significantly different at p ≤ 0.05.Values followed by asterix indicate the regrowth of viable colony cells. < is below the detection limit.

Table S4. Time-kill kinetics antimicrobial study of Z. usambarense extract against *Enterococcus faecalis*

| Incubation time (Hour) | Mean bacterial population  (CFU/mL) | | | | Percentage reduction | | | Log reduction | | |
| --- | --- | --- | --- | --- | --- | --- | --- | --- | --- | --- |
|  | 0.5×MIC | 1×MIC | 2×MIC | Control | 0.5×MIC | 1×MIC | 2×MIC | 0.5×MIC | 1×MIC | 2×MIC |
| 0 | 1.90×10^6^±2.00^a^ | 1.68×10^6^±2.00^a^ | 1.58×10^6^±2.00^a^ | 1.90×10^6^±2.65^a^ | - | - | - |  |  |  |
| 2 | 1.00×10^6^±2.65^a^ | 9.80×10^5^±2.00^b^ | 8.87×10^5^±0.58^b^ | 2.02×10^6^±2.08^a*^ | 47.4 | 41.7 | 43.9 | 0.28 | 0.23 | 0.25 |
| 4 | 1.42×10^6^±1.00^*^ | 1.40×10^6^±1.15^*^ | 4.07×10^5^±1.15^c^ | 2.55×10^6^±1.53^*^ | 25.3 | 16.5 | 74.3 | 0.13 | 0.08 | 0.59 |
| 6 | 1.60×10^6^±1.00^*^ | 1.76×10^6^±3.21^*^ | 6.87×10^5^±0.58^*^ | 2.77×10^6^±2.52^*^ | 15.8 | 4.96 | 56.5 | 0.08 | -0.02 | 0.36 |
| 8 | 2.88×10^6^±3.06^*^ | 2.84×10^6^±4.00^*^ | 9.87×10^5^±0.58^*^ | 2.97×10^6^±2.89^*^ | -51.4 | -69.0 | 37.6 | -0.18 | -0.22 | 0.21 |
| 24 | 2.92×10^6^±2.08^*^ | 3.04×10^6^±3.06^*^ | 1.74×10^6^±5.50^*^ | 3.08×10^6^±2.52^*^ | -53.7 | -81.0 | -10.6 | -0.19 | -0.25 | -0.04 |

Values are means ± standard deviation of three replicates. Values in each column followed by different superscript within each column are significantly different at p ≤ 0.05.Values followed by asterix indicate the regrowth of viable colony cells.

Table S5.Time-kill kinetics antimicrobial study of Z. usambarense extract against *Escherichia coli*

| Incubation time (Hour) | Mean bacterial population  (CFU/mL) | | | | Percentage reduction | | | Log reduction | | |
| --- | --- | --- | --- | --- | --- | --- | --- | --- | --- | --- |
|  | 0.5×MIC | 1×MIC | 2×MIC | Control | 0.5×MIC | 1×MIC | 2×MIC | 0.5×MIC | 1×MIC | 2×MIC |
| 0 | 2.50×10^6^±1.00^a^ | 1.99×10^6^±1.00^a^ | 1.80×10^6^±1.53^a^ | 2.50×10^6^±2.00^a^ | - | - | - |  |  |  |
| 2 | 1.32×10^6^±7.64^a^ | 1.12×10^6^±7.64^a^ | 1.02×10^6^±7.64^A^ | 2.82×10^6^±1.53^a*^ | 47.2 | 43.7 | 43.3 | 0.28 | 0.25 | 0.25 |
| 4 | 1.03×10^6^±2.89^a^ | 9.67×10^5^±7.64^b^ | 9.57×10^5^±4.04^b^ | 3.04×10^6^±3.05^*^ | 58.8 | 51.4 | 46.8 | 0.39 | 0.31 | 0.28 |
| 6 | 1.95×10^6^±5.00^*^ | 1.80×10^6^±5.00^*^ | 5.50×10^5^±5.00^c^ | 3.35×10^6^±5.00^*^ | 22.0 | 9.55 | 69.4 | 0.11 | 0.04 | 0.52 |
| 8 | 2.85×10^6^±4.58^*^ | 2.85×10^6^±5.03^*^ | 1.57×10^6^±2.52^*^ | 3.60×10^6^±1.15^*^ | -14.0 | -43.2 | 12.8 | -0.05 | -0.15 | 0.06 |
| 24 | 3.22×10^6^±2.52^*^ | 3.41×10^6^±2.65^*^ | 2.67×10^6^±7.64^*^ | 3.76×10^6^±5.14^*^ | -28.8 | -71.4 | -48.3 | -0.11 | -0.23 | -0.17 |

Values are means ± standard deviation of three replicates. Values in each column followed by different superscript within each column are significantly different at p ≤ 0.05.Values followed by asterix indicate the regrowth of viable colony cells.
